# Relearning Upper Limb Proprioception After Stroke Through Robotic Therapy: A Feasibility Analysis

**DOI:** 10.3390/jcm14072189

**Published:** 2025-03-23

**Authors:** Ananda Sidarta, Yu Chin Lim, Christopher Wee Keong Kuah, Karen Sui Geok Chua, Wei Tech Ang

**Affiliations:** 1 Rehabilitation Research Institute of Singapore, Lee Kong Chian School of Medicine, Nanyang Technological University, Singapore 308232, Singapore; 2 National Neuroscience Institute, Tan Tock Seng Hospital, Singapore 308433, Singapore; 3 Institute of Rehabilitation Excellence, Tan Tock Seng Hospital Rehabilitation Centre, Singapore 307382, Singapore; 4 School of Mechanical and Aerospace Engineering, Nanyang Technological University, Singapore 639798, Singapore

**Keywords:** proprioception, sensory, stroke, robotics, reward, neurological rehabilitation

## Abstract

**Background:** Motor learning can occur through active reaching with the arm hidden from view, leading to improvements in somatosensory acuity and modulation of functional connectivity in sensorimotor and reward networks. In this proof-of-principle study, we assess if the same paradigm benefits stroke survivors using a compact end-effector robot with integrated gaming elements. **Methods:** Nine community-dwelling chronic hemiplegic stroke survivors with persistent somatosensory deficits participated in 15 training sessions, each lasting 1 h. Every session comprised a robotic-based joint approximation block, followed by 240 repetitions of training using a forward-reaching task with the affected forearm covered from view. During movement, the robot provided haptic guidance along the movement path as enhanced sensory cues. Augmented reward feedback was given following every successful movement as positive reinforcement. Baseline, post-intervention, and 1-month follow-up assessments were conducted, with the latter two sessions occurring after the final training day. **Results:** Training led to reliable improvements in endpoint accuracy, faster completion times, and smoother movements. Acceptability and feasibility analyses were performed to understand the viability of the intervention. Significant improvement was observed mainly in robotic-based sensory outcomes up to a month post training, suggesting that training effects were predominantly sensory, rather than motor. **Conclusions:** The study outcomes provide preliminary evidence supporting the feasibility of this intervention for future adoption in neurorehabilitation.

## 1. Introduction

Motor impairment, primarily in the form of hemiparesis, is the most common disability following a stroke. In addition to motor deficits, stroke survivors typically suffer from a range of impairments across different somatosensory sub-modalities [1,2], including diminished touch perception, compromised pressure and sharpness sensation, reduced ability to sense temperature, and deficits in proprioception. Among these, proprioception plays a pivotal role in enabling co-ordinated and smooth movements by providing information about the position, motion, and force generated by different body parts in space [3]. When proprioception is compromised, stroke survivors struggle to estimate their limb position and maintain a steady posture in the presence of poor vision or neglect [4,5]. Those with upper limb somatosensory impairment often report challenges in performing gross and fine motor activities daily [6,7]. While there is evidence of spontaneous recovery in the somatosensory domain [8], some continue to live with persistent somatosensory deficits [9,10].

Recent work has highlighted the interdependency between somatosensory and motor recovery, opening a fresh outlook into sensory-focused intervention post-stroke [11,12]. Despite implementation barriers, somatosensory recovery has slowly gained increasing recognition as an essential factor in a more holistic rehabilitation after stroke [13,14,15,16]. Stroke patients with upper limb sensory loss often do not receive targeted treatment, as therapists typically rely on everyday activities rather than sensory-specific retraining [17]. Robot-assisted rehabilitation targeting proprioception is arguably important for improving sensorimotor functions post-stroke. A recent systematic review suggests that proprioceptive training shares similar characteristics with motor learning [18]. Various approaches of proprioceptive retraining have been proposed depending on which limbs are involved (refer to [19,20] for a review). Passive training can be suitable for those with severe motor impairment, but it may induce boredom. In contrast, the active training mode allows stroke survivors to interact with a robotic device to execute some task-specific movements and receive appropriate real-time performance feedback [21,22,23]. Tasks that simultaneously target both motor and sensory aspects are thought to enhance somatosensory relearning while simultaneously improving mobility in the affected upper limb [24].

The main aim of this paper is to demonstrate a new robotic upper limb training paradigm that targets both proprioceptive and motor functions in stroke survivors with persistent somatosensory impairment. Key features of the paradigm include (*i*) a compact and portable robotic device that allows two-dimensional planar movement, (*ii*) a proprioceptive neuromuscular technique via joint approximation involving compression of the elbow and shoulder joints, (*iii*) sensorimotor learning through reaching tasks with forearms covered from view, which are repetitive and specific to elbow and shoulder joints, and (*iv*) multiple forms of augmented reward feedback to boost performance. It was hypothesised that this approach would improve position sense (sensory domain), which translates to movement accuracy and functional domains at the same time.

## 2. Materials and Methods

### 2.1. Study Design

**Figure 1:**
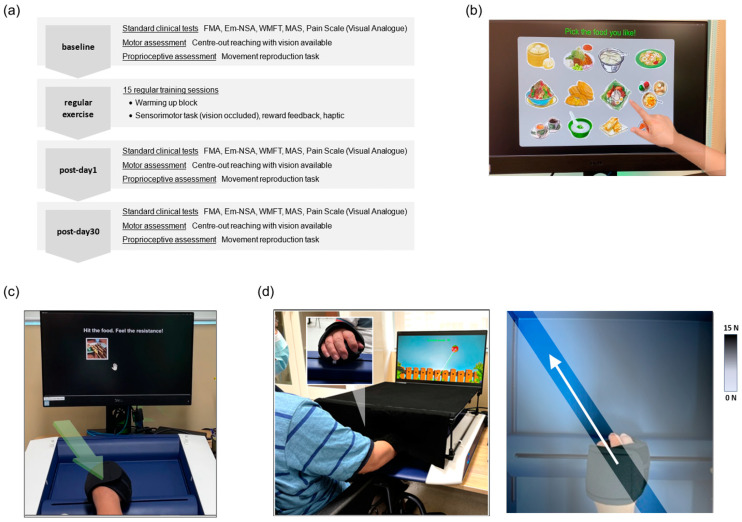
(**a**) Block diagram of the study, comprising a baseline assessment, 15 training sessions, and 2 post-training assessments; (**b**) in the first warm-up block, the accompanying therapist pointed at an item on the LCD screen, prompting the participant to move the robotic handle toward it; (**c**) the second warm-up block involved the joint approximation task, where participants moved the handle from a start position to a designated location while encountering a position-dependent resistive force (green arrow), which they were instructed to observe; (**d**) during training, participants performed outward reaching movements while their affected arm was occluded by an arm cover. The inset illustrates the front view of the hand gripping the robotic handle. The next diagram on the right shows an example of a virtual channel while the participant is reaching 120° towards the left.

This was a prospective open-label feasibility trial conducted between 2020 and 2022 in two day-rehabilitation clinics in Singapore. All participants provided written informed consent prior to the research interventions. This study used the H-Man robot (Articares Pte Ltd., Singapore) [25], a 2D rehabilitation robotic device placed on a height-adjustable table, with a 33 cm × 34 cm workspace. The device had a handle resembling an ergonomic computer mouse that was designed for this rehabilitation study (Figure 1d). The X and Y co-ordinates of the handle were derived from the encoders in the robot. While seated with their affected hand fastened to the handle, participants could see an LCD display showing some game interface written in MATLAB R2019b (MathWorks, Inc., Natick, MA, USA).

### 2.2. Study Participants

Community-dwelling stroke survivors were recruited via convenience sampling. Participants were enrolled if they fulfilled the following inclusion criteria: (1) had first-ever ischemic or haemorrhagic stroke diagnosed by neurologists and confirmed by neuroimaging, (2) were aged 21–75 years, (3) had a duration of >6 months post-stroke, (4) had sensory impairments as assessed using the Erasmus MC modifications of the Nottingham Sensory Assessment (Em-NSA) with scores ≤ 6 out of 8 in at least two categories, (5) had hemiparetic weakness with the presence of minimum shoulder abduction and elbow flexion strength grade ≥ 2 according to the Medical Research Council (MRC) scale, and (6) were able to use the affected arm to move an item forward >5 cm. The exclusion criteria were as follows: (1) bilateral upper limb motor impairment from central, spinal, or peripheral nerve causes; (2) shoulder and elbow spasticity, as assessed by the Modified Ashworth Scale for Spasticity >2; (3) unilateral neglect, as assessed by the Star Cancellation Test (score < 44); (4) cognitive impairment (memory, attention, and language), as examined by the Mini-Mental State Examination (scores < 25); (5) a known medical history of mental disorders; and (6) a visual analogue scale (VAS) for pain > 5 out of 10.

### 2.3. Study Protocol

Eligible participants completed a baseline assessment, followed by 15 sessions of robotic-based intervention and post-training assessments, as illustrated in Figure 1a. Each gamified intervention lasted for approximately an hour, conducted 3 days per week over 5 weeks, and was always attended by a trained therapist. The session started with a warm-up exercise, joint-approximation exercise, followed by sensorimotor training. In the initial session, some practice trials were provided to help the participants understand each task. The therapist also maintained a diary containing the participant’s comments, incidental observations, or unforeseeable adverse events.

(1) Warm-up and resistive exercise

The warm-up phase began with a functional reaching task, where participants moved their affected arm toward a specified food item shown on the screen as selected by the therapist (Figure 1b). The vision of the paretic arm was not occluded. This was followed by 16 repetitions of the approximation technique targeting the elbow and shoulder joints. While moving the handle 10 cm towards a target, the robot produced a spring-like resistive force (stiffness = 900 N/m), opposing the movement (Figure 1c). This resistance was position-dependent, i.e., it increased as the distance between the handle and the target location increased. Participants were instructed to pay attention to the sensation of this resistive force. After a brief break, the therapist assessed the participants’ readiness to proceed with the training.

(2) Sensorimotor training task

The sensorimotor training setup is shown in Figure 1d. The affected forearm was occluded using a custom rectangular box, eliminating any visual feedback from the upper limb. On the LCD screen, a gaming interface displayed a start point and a handle position in space. This handle cursor disappeared beyond a 2.0 cm distance from the start to encourage reliance on proprioception. Four equidistant visual targets appearing as 3.0 cm diameter cartoon characters were established 15 cm from the start point (30°, 60°, 120°, and 150°). Targets were presented sequentially, alternating between a 30° to 150° and 150° to 30° order across blocks. After a movement initiation cue, participants commenced the forward-reaching motion as straight and directly as possible within 5.0 s. When the movement had been completed, reward feedback was provided so that they understood their performance accuracy. The robot subsequently moved the hand back to the start along a straight and smooth path (stiffness = 3500 N/m, damping = 20 N.s/m), and the next trial continued. Each session had 10 blocks of 24 repetitions, with its own gaming theme per block to prevent boredom. A brief pause was provided to avoid fatigue and to adjust the handle’s hand grip if necessary. Stretching and gentle massage of the paretic shoulder and elbow were performed occasionally to help the participants relax and lower their muscle tone. The participant’s trunk was not restrained to a chair, but the therapist was present to limit any apparent trunk movement during the task.

(3) Augmented feedback

As the participants performed the movement, online haptic feedback in the form of a “virtual channel” connecting the start position and the target was produced by the robot handle (stiffness = 1000 N/m), as shown in Figure 1d. This mechanism applied a spring-like force whenever the movement deviated excessively from the intended path, effectively serving as an enhanced error signal to keep the handle on track. However, no virtual channel was applied in the radial direction, requiring participants to learn movement control and avoid overshooting independently. Reward feedback is useful in rehabilitative training as a motivational boost by rewarding successful performance [26]. Here, two types of terminal feedback were given after each movement to indicate the participant’s performance. The first feedback appeared visually as a 2D movement trajectory, shown together with a reference line connecting the start position and the target. This trajectory and the reference line were still given in the case of an incorrect or unsuccessful trial to improve subsequent performance. Another terminal feedback was provided as a pleasant audio tone after every successful movement outcome as a reward. A successful movement was judged based on two criteria, i.e., if the endpoint error and lateral perpendicular deviation were within the span of the target (≤3.0 cm), which remained fixed across training sessions. Intermittently, an audio message was played at random intervals by the computer after successful (e.g., “Good job!”) or unsuccessful (e.g., “Keep trying!”, “Don’t give up”, or “Move straighter and closer”) trials, respectively.

### 2.4. Assessment Outcomes

(1) Robotic-based assessments

Robotic-based assessments were carried out as the primary outcome measures, comprising one motor test and sensory test (i.e., movement reproduction and position-matching). Each test had one block of 24 trials. In the motor test, participants moved the robotic handle with their affected arm to a visual target, 1.5 cm in diameter, replicating the sensorimotor training task but without any occlusion of the arm or augmented feedback available. Following the motor task, proprioception and kinaesthesia were assessed by the movement reproduction task. Here, the ability to passively sense one’s own movement was evaluated by first perceiving the movement of the affected arm produced by the robot and subsequently reproducing that same movement accordingly.

(2) Standard clinical scales

Three ordinal clinical scales were used to evaluate the effects of robotic-based training as secondary outcome measures. The Erasmus-modified of the Nottingham Sensory Assessment (Em-NSA) was used to evaluate upper limb sensory impairments, such as light touch, tactile discrimination, and proprioception [27]. Overall upper limb functional ability was assessed using the Streamlined Wolf Motor Function Test (WMFT), which comprises six timed tasks: hand-to-table, hand-to-box in front, lifting a can, lifting a pencil, folding a towel, and reach and retrieve [28]. Task performance was evaluated using movement time (MT) and functional ability score (FAS). Finally, the level of motor impairment was evaluated using the Fugl-Meyer Assessment for Upper Extremity (FMA-UE) [29]. All clinical assessments were conducted by a certified therapist not involved in the regular intervention. Clinical outcomes were compared with the minimum clinically important difference (MCID). To the best of our knowledge, there is no study reporting the MCID for the Em-NSA in chronic stroke. The MCID reference value for the WMFT and for the FMA-UE is based on prior studies [30,31,32].

### 2.5. Data Analyses and Statistical Tests

The robotic device recorded 2D reaching kinematics, with the raw data filtered using a 4th-order Butterworth lowpass filter (cut-off frequency: 20 Hz) and processed using MATLAB R2019b. Training gain was achieved when there was accuracy improvement as a result of the intervention, which was quantified using endpoint error (in cm), i.e., the Euclidean distance between the actual target centre and the position that the participant felt to be the target location. Within-session performance was represented by average accuracy across all blocks of each session. A linear fit was subsequently performed for each participant over the 15 sessions to estimate the training gain, where a negative slope (reduction in reaching error) indicated an improvement in accuracy. In addition, to check whether movement smoothness or quality of reaching movement improved after training [33,34], we computed the *spectral arc length* (SPARC) using the speed information. This metric is a dimensionless quantity that has been verified and used in stroke studies (for a review, see [35]). Its numerical value is negative, such that the greater the value (i.e., less negative) is, the smoother the movement is. For the motor and sensory tests, endpoint error was again used as the primary measure of accuracy. In the sensory tests, this metric represents how well the participants sensed the end position of the reference movement.

Nonparametric statistical tests, including the Wilcoxon signed-rank test and Spearman’s rho, were used for small sample data. The aligned-rank transform (ART) with mixed-effects modelling [36,37], a nonparametric alternative to analysis of variance (ANOVA), was used to compare differences across training sessions, as well as across assessment timepoints. The mean (M) ± standard deviation (SD) and median of each performance measure were reported. Average differences in assessment outcomes before and after training were presented as a 95% confidence interval estimated using the nonparametric bootstrap method with 1000 repetitions [38], without relying on the normality assumption. All the statistical analyses were performed in R version 4.0.4. Statistical significance was taken at α-level = 0.05.

### 2.6. Analysis of Feasibility

A feasibility analysis was conducted to evaluate the practicality of the proposed robotic-based training paradigm in stroke rehabilitation. First, the perception of the participating stroke clients was assessed using a short questionnaire consisting of seven Likert scale questions, which was administered after the last training session. This questionnaire aimed to obtain opinions from the participants on the robotic-based intervention they had just completed. The first two questions assessed the overall enjoyment of the training setup, and the next two dealt with the perceived benefits of the session as an intervention programme. The last four questions examined the role of reward feedback and the virtual wall. Notes or comments from the participants or any incidental observations were reported. Information related to recruitment, trial scheduling, tolerability and adherence, adverse events, and training gain or outcome measures were included as the indicators for feasibility [39]. The information would then be used to compile the strengths, weaknesses, opportunities, and drawbacks of the proposed stroke intervention.

## 3. Results

A total of 24 community-dwelling chronic stroke survivors were screened from local community rehabilitation clinics, of whom only 9 fulfilled the eligibility criteria and completed the study without any adverse events (Table 1). On the intervention day, eligible participants only completed the robotic-based training, replacing any other therapy the persons normally had in the clinic.

### 3.1. Assessment Outcomes

(1) Robotic Assessments

Robotic-based tests created behavioural performance indices that measured training-related effects in the motor and proprioceptive domains. For the motor test, the mean endpoint error decreased from 1.49 ± 1.02 cm (median = 1.46) to 1.33 ± 1.54 cm (median = 0.91) post-training, and then to 1.17 ± 0.82 cm (median = 0.91) 1 month later, although the mean reduction was not significant (F(2,16) = 1.09, *p* = 0.10, η^2^_P_ = 0.11). On the other hand, improvement in proprioception was observed during the movement reproduction test (F(2,16) = 3.61, *p* < 0.01, η^2^_P_ = 0.47). Specifically, a gain was observed from the baseline (7.27 ± 2.23 cm (median =6.59)) to postintervention (5.59 ± 1.87 cm (median = 5.07)) (corrected *p* = 0.02) and to 1-month follow-up (5.66 ± 2.46 cm (median = 4.84)) (corrected *p* = 0.01). The completion time and smoothness before and after training for both tests were similar, with no significant differences.

(2) Standard Clinical Scales

**Figure 2:**
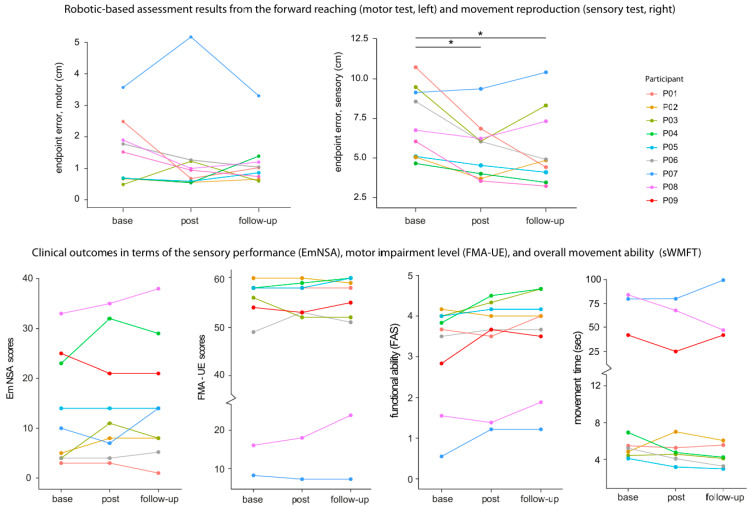
Changes in robotic assessment indices of motor and sensory tests, and upper limb clinical scores for the sensory (Em-NSA), motor impairment (FMA-UE), and functional ability (functional ability score—FAS and movement time of the streamlined WMFT), before and after training for each participant. The higher the ordinal scores, the better the performance outcome. As for the MT, a smaller number (in seconds) means better improvement. The * in the top panel denotes *p* < 0.05.

Table 2 and Figure 2 show the magnitude of change after training in each participant compared to baseline. Sensory improvement just after training was noted in four participants (P02, P03, P04, and P08). The overall difference was not significant albeit there was an average increase (F(2,16) = 1.35, *p* = 0.28, η^2^_P_ = 0.14). Improvement in post-training was found in six participants for the WMFT. Specifically, four participants improved beyond the MCID in FAS (P03, P04, P07, and P09) and three in MT (P04, P08, and P09). The overall FAS difference reached a trend towards significance (F(2,16) = 3.12, *p* = 0.07, η^2^_P_ = 0.28). However, the overall MT difference did not differ significantly from the baseline (F(2,16) = 1.69, *p* = 0.20, η^2^_P_ = 0.17). Improvement in FMA-UE was found in three participants (P04, P06, and P08), but the magnitude was smaller than the MCID. The overall difference was not significant in post-training and during follow-up (F(2,16) = 0.31, *p* > 0.50, η^2^_P_ = 0.03).

### 3.2. Training Performance

**Figure 3:**
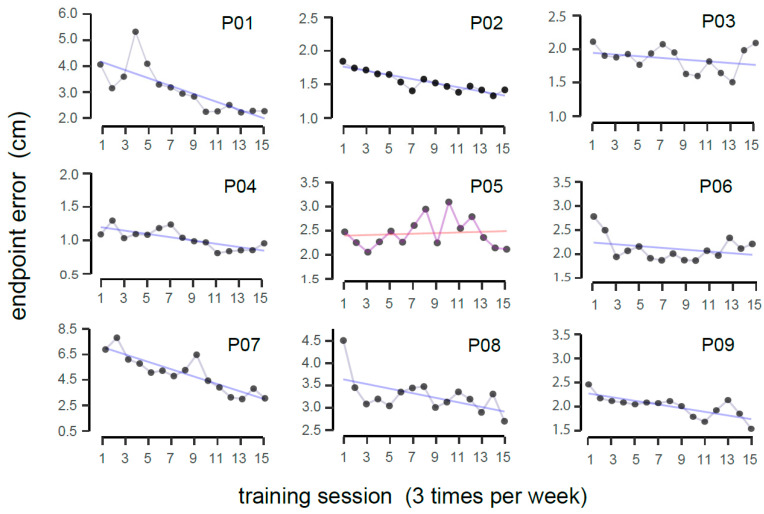
Deviation between the actual and perceived target locations during training, with Pxx representing individual participants. A linear fit was applied to the data points, where the purple line indicates a reduction in endpoint error (signifying improved movement accuracy), and red line denotes no obvious improvement over time.

Performance during the training sessions was represented by the endpoint error, completion time, and smoothness. Figure 3 depicts the progression of accuracy performance over time as denoted by the endpoint error (in cm). For every participant, each data point represents an average endpoint error of a single session across all blocks. The blue line represents a linear fit, where a downward trend indicates a reduction in reaching error to signify beneficial intervention. On average, the slope was found to be negative (average slope = −0.068 ± 0.1 cm/session), suggesting an improvement in accuracy over training sessions (Wilcoxon signed-rank test, V = 1.0, *p* = 0.008; r_effect_ = 0.84). In conjunction, a linear fit was also applied to the average number of rewarded trials across different sessions, and an upward trend was found (average slope = 0.007 ± 0.01/session). This means that the participants on average made increasingly more successful trials over time (Wilcoxon signed-rank test, V = 45, *p* = 0.004, r_effect_ = 0.89).

**Figure A1:**
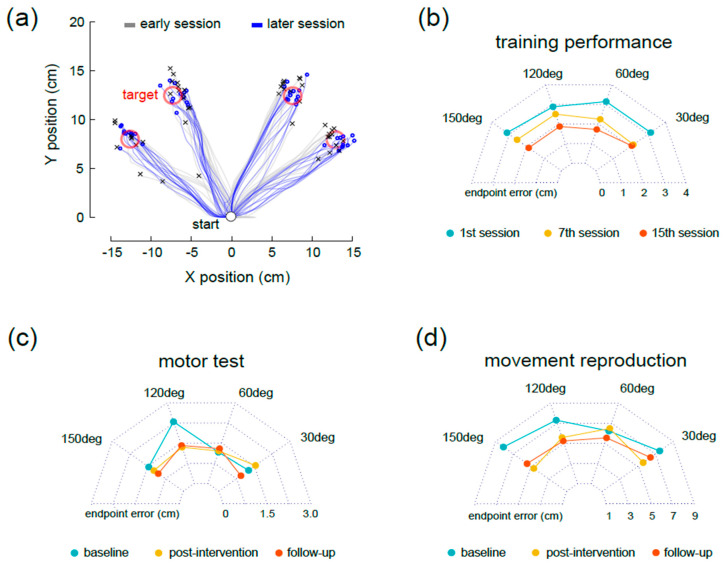
Panel (**a**) shows typical reaching patterns of a representative stroke participant during the first and last training sessions; (**b**–**d**) depict the group average (*n* = 9) of the endpoint error in each reaching direction, during training, motor, and sensory tests.

To explore the data deeper rather than using a fitted straight line, a two-way nonparametric repeated-measures test was conducted on the endpoint error, with the session and target location as the factors. There was a significant main effect of session (F(14,112) = 3.14, *p* < 0.001, η^2^_P_ = 0.28), but no interaction effect was observed. Accuracy performance for the different reaching directions during training and assessments is presented in Appendix A, Figure A1. Post hoc pairwise comparisons with Tukey’s correction revealed that the most reliable reduction in error started from the ninth session (the mean errors for the 1st, 9th, and 15th sessions were 2.98 ± 2.87 cm (median = 2.04), 2.35 ± 2.20 cm (median = 1.73), and 1.88 ± 1.25 cm (median = 1.62), respectively; corrected *p* < 0.05). A similar session effect was found concerning the smoothness metric, SPARC (F(14,112) = 2.48, *p* < 0.01; η^2^_P_ = 0.23).

### 3.3. Acceptability and Feasibility Analyses

**Figure 4:**
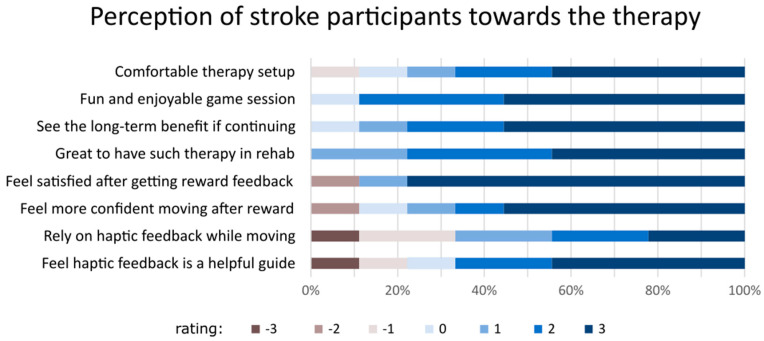
The perception of stroke participants about the training sessions they attended using a Likert scale questionnaire. The rating ranges from 3 (the most agreeable) to −3 (least agreeable).

From the acceptability questionnaire, the majority of the participants expressed that robotic-based training was fun and comfortable and was also viewed as useful in the long term if the programme was to continue being available for them (Figure 4). Three participants (P01, P03, and P05) expressed interest in continuing therapy beyond the study period. Overall, there was a consensus among the participants wanting the rehabilitation clinics to offer this type of therapy. However, two participants (P05 and P06) held less favourable views regarding the provision of reward feedback for successful movements and the use of the haptic channel to guide movement. Indicators for feasibility analysis are presented in Table 3, comprising eight different categories.

## 4. Discussion

Voluntary movement and motor learning rely on intact somatosensory feedback. Before reaching for an item, the brain uses feedforward control of a forward model to create an effective motor plan and continuously fine-tunes motor performance using proprioceptive feedback. However, these signals are commonly impaired following a stroke. This study presents a repetitive exercise with a compact planar robot to retrain upper limb proprioception while simultaneously promoting active use of the affected limb. The training paradigm incorporates key elements of rehabilitation, namely, the joint approximation technique using the robot’s resistive mode, forward planar reaching tasks with the forearm blocked from view, and augmented feedback to enhance training performance. Although most daily activities involve 3D movements, planar robotic therapy remains valuable for relearning fundamental movement patterns (e.g., elbow flexion/extension) in a more task-specific and controlled environment. It is also suitable for severely impaired stroke survivors who cannot reach upward against gravity.

Interventions using sensory stimulation have garnered recognition in modern neuro-rehabilitation. Techniques such as intermittent compression or pressure and limb vibration [20,40,41,42] have shown promising results in improving sensory and motor functions after stroke. Joint approximation, a facilitatory technique that induces co-contraction of the muscles surrounding the joints of the affected upper limb, promotes the activities of muscle spindles and Golgi tendon organs to improve functional outcomes [43,44]. This can be achieved by pushing against a wall or resistance, causing concentric muscle contraction in the upper limb. While, traditionally, joint approximation was administered by the therapist directly, this study applied the technique using a rehabilitation robot at the beginning of every training.

Prior research has focused predominantly on the sensory sub-modalities of upper limb distal joints [45,46,47,48], but this study focused on the proximal joints (elbow and shoulder). The upper limb tasks we used actively engaged the affected upper limb, unlike some prior studies that used purely passive sensory exercise [49]. A recent systematic review [24] suggested that exercises that synchronously combine motor and proprioceptive retraining tend to foster stronger connections between sensorimotor cortical regions, thereby promoting increased neuroplasticity in associated areas, than does a paradigm in which training tasks are implemented sequentially. In this study, the reaching task closely resembled the one employed in prior research involving young adults, which is capable of inducing cortical changes in the sensorimotor and reward networks [50,51]. The distinguishing features as compared to those studies include gamification with four reaching directions and enriched augmented feedback involving a virtual wall during training.

Our intervention aligns with established principles of motor learning, as found in the literature (for a comprehensive review, please refer to [52]). The 1 h training sessions were carried out three times a week for five weeks, comprising 3600 forward-reaching movements. In every block, the target appeared six times in sequence, starting from 30° to 150° or from 150° to 30°. This task paradigm is consistent with massed practice (i.e., repetitive without breaks) and spaced practice principles (i.e., having a break in between two consecutive training sessions), which are important for motor learning and memory consolidation. The provision of terminal feedback and haptic feedback during reaching emulates the importance of feedback in motor learning. Exercising with the same robotic device typically includes some degree of robotic assistance, which can yield measurable changes in performance [53]. However, our training task excluded assistance because participants had adequate muscle strength. The current training paradigm employed a uniform difficulty level (i.e., same target size) across stroke participants to obtain a preliminary understanding of their interaction with the task. To introduce greater challenges and enhance engagement in the rehabilitation process, a variable level of difficulty with a varying reward gradient can be employed with the task. This variation can be tailored for different individuals to achieve more personalised training, a trend in robot-assisted rehabilitation that has grown in recent years [54,55].

Overall, nine eligible chronic stroke survivors showed reliable training gains, as indicated by the gradual reduction in endpoint error (Figure 3). The mean absolute error dropped to about 2.0 cm, comparable to that of healthy adults in a prior study using a similar training paradigm (1.0 to 1.5 cm range) [50]. The robotic-based tests included a forward-reaching task with the arm visible (motor test) and a movement reproduction task with eyes closed (sensory test). Our version of the sensory test required the participants to reproduce the movement with the same affected arm, unlike some past studies that use the less affected arm. Reliable improvements after training (i.e., during post-training and 1-month follow-up tests) were limited to the sensory test, as the reduction in reaching accuracy of the motor test was not statistically significant. This suggests that the training benefit is mainly in the sensory rather than motor domain. Our finding is also consistent with past research using a wrist robot on 12 patients [21]. But this result shall be interpreted with caution. During the robotic motor test, participants reached to a target with their view of the affected arm available. It is possible that the motor impairment was not as bad, such that the capacity to improve is limited. This trend is evident in Figure 2 (top left panel), where the average error closely matches that of healthy adults (1.0 to 1.5 cm range). In terms of clinical outcome measures, some participants showed improvement beyond the MCID. However, average changes in the scores were not statistically significant. The lack of significant changes in the clinical scores is likely due to the nature of the clinical scales, which are ordinal and known to be subjective and less sensitive. Recent studies have proposed modern technology for physiological and kinematic assessments, which are more sensitive and objective and less prone to ceiling effects [56,57].

Acceptability and feasibility were reported and summarised into eight categories. Overall, the participants viewed the training programme favourably. Our recent survey of current clinical practices has indicated that most clinicians view proprioceptive training as an integral part of rehabilitation [16]. As such, portable and compact robotic devices can provide a more objective and standardised tool to assess and retrain the sensorimotor system post-stroke. In addition, the training protocol was deemed acceptable, not too strenuous, and no adverse events occurred. One notable observation is that another person had to accompany the participants in the intervention. This is due to instances in which the hand slipped off the grip while the arm cover was installed. Participants were unable to realise this themselves due to their somatosensory deficits, thus possibly disrupting the training process. As such, the suitability of this training in a minimally supervised telerehabilitation setting requires further research.

This study had some limitations. Primarily, it was a single-arm study with a small sample size. Although training gains were observed over 15 sessions, only nine chronic cases with persistent somatosensory deficits were involved. This limited sample size may have contributed to the lack of significant improvement in clinical scores across the board. Additionally, the absence of randomisation and placebo evaluation limits the strength of the evidence and the proof of concept, preventing generalisation of the results. Therefore, a future randomised controlled trial (RCT) with a control group undergoing conventional proprioceptive training is warranted to generalise these findings. Further investigation is also needed to determine the extent of proprioceptive enhancement required for stroke survivors to experience noticeable improvements in daily life.

## 5. Conclusions

The robot-assisted intervention presented here produced training gains based on the endpoint accuracy measure. Significant improvement was observed in the movement reproduction test (sensory) and not in the motor test. On average, limited changes in clinical scales were noted with some variability, and not all participants improved beyond the MCID. This study serves as a proof of concept and provides insights into the feasibility of the proposed robotic-based therapy tool for community-dwelling stroke survivors with somatosensory deficits.

## Figures and Tables

**Table 1:** Baseline demographic and clinical characteristics of participants. All participants were right-handed, with an average duration post-stroke of 3.56 ± 1.94 years.

Case No.	Gender	Age	Duration of Stroke	Nature of Stroke	Affected Limb
P01	Male	65	4 years	Haemorrhagic	Right
P02	Male	61	4 years	Haemorrhagic	Right
P03	Male	71	3 years	Haemorrhagic	Left
P04	Female	38	3 years	Haemorrhagic	Right
P05	Male	39	2 years	Haemorrhagic	Right
P06	Male	57	1 year	Haemorrhagic	Right
P07	Male	63	7 years	Ischemic	Left
P08	Female	74	6 years	Ischemic	Right
P09	Male	58	2 years	Ischemic	Left

**Table 2:** Changes in clinical scores after training are reported as mean differences with respect to the baseline, where green colour denote improvement. Sensory, functions, and motor impairment scales are based on the standardised tests (see main text): Em-NSA, WMFT, and FMA-UL.

Case No.	Sensory (Post)	Sensory (Follow-Up)	Functions (Post)	Functions (Follow-Up)	Task Time (Post)	Task Time (Follow-Up)	Impairment (Post)	Impairment (Post)
P01	0	−2	−0.17	**0.33**	**−0.22**	0.08	0	0
P02	**3**	**3**	−0.17	−0.17	2.17	1.21	0	−1
P03	**7**	**4**	**0.33**	**0.67**	0.15	**−0.32**	−4	−4
P04	**9**	**6**	**0.67**	**0.83**	**−2.18**	**−2.70**	**1**	**2**
P05	0	0	**0.17**	**0.17**	**−0.93**	**−1.13**	0	**2**
P06	0	**1**	**0.17**	**0.17**	**−1.14**	**−1.96**	**4**	**2**
P07	−3	**4**	**0.67**	**0.67**	0.15	19.78	−1	−1
P08	**2**	**5**	−0.17	**0.33**	**−16.44**	**−37.12**	**2**	**8**
P09	−4	−4	**0.83**	**0.67**	**−17.22**	0	−1	**1**
Mean diff	1.56 (−1.22, 4.11)	1.89 (−0.11, 4.11)	0.26 (0.02, 0.50)	0.41 (0.22, 0.61)	−3.96 (−7.92, 1.08)	−2.46 (−10.93, 7.63)	0.22 (−1.11, 1.56)	1.00 (−1.00, 2.89)
MCID			0.20	0.20	−1.50	−1.50	7.25	7.25

Values indicated in bold denote improvement in outcome measures. MCID = minimum clinically important difference. Numbers inside the brackets represent 95% confidence interval of mean difference calculated through bootstrapping.

**Table 3:** Indicators adopted in this study to evaluate the feasibility of the proposed therapy.

Indicator	Information
Recruitment	Screening was subject to COVID-19 restrictions. Of the 24 stroke clients, only 10 (41.66%) met the inclusion criteria, though 1 declined to enrol. Among those ineligible, 14 (58.33%) either did not exhibit any sensory deficits or were excluded due to excessive shoulder pain, insufficient muscle strength, or poor cognitive function.
Trial scheduling	All participants made their best effort to attend therapy sessions three times per week. However, as community-dwelling clients, their schedules were occasionally disrupted by routine commitments. Sessions were rescheduled due to factors such as vaccinations, medical checkups, or feeling unwell.
Tolerability and adherence	The study procedures were well tolerated by all participants. No adjustment to the therapy protocol was necessary, as each of the 10 blocks was sufficiently brief and manageable. One participant (P05) experienced frustration with failed trials early in training and reported some sleep disturbances. However, all participants successfully completed all sessions without any dropouts.
Manpower	Two personnel were required for the study. During training, the study team noted that participants occasionally failed to notice their hands slipping off the handle. To manage this, the accompanying therapist checked the grip through the side gap of the arm cover after each training block, ensuring proper hand positioning. This shows that a minimum of one trained personnel was necessary for the intervention. A separate therapist not involved in the training conducted the baseline, post-training, and follow-up assessments.
Adverse events	No adverse events, physical injury, or death were reported.
Training gain	A positive training gain was seen in each individual (see main text and Figure 3), except for P05.
Assessment outcomes	The clinical data showed a degree of variability, where not all participants improved beyond the MCID. In contrast, the robotic-based measure of sensory performance, considered to be more sensitive, showed a significant difference after training (see main text and Figure 2). These improvements were not observed in the motor test, suggesting that the primary benefit of the training is sensory rather than motor.
Perception of participants	This is reflected in the result of the acceptability questionnaire (see main text and Figure 4 below).

## Data Availability

Data are available upon a reasonable request to the corresponding author.

## Appendix A

Figure A1 shows additional results related to the 2D reaching kinematics employed in this study with four different planar directions.
